# Sampling strategies for monitoring and evaluation of morbidity targets for soil-transmitted helminths

**DOI:** 10.1371/journal.pntd.0007514

**Published:** 2019-06-26

**Authors:** Federica Giardina, Luc E. Coffeng, Sam H. Farrell, Carolin Vegvari, Marleen Werkman, James E. Truscott, Roy M. Anderson, Sake J. de Vlas

**Affiliations:** 1 Department of Public Health, Erasmus MC, University Medical Center Rotterdam, Rotterdam, The Netherlands; 2 London Centre for Neglected Tropical Disease Research (LCNTDR), Department of Infectious Disease Epidemiology, Imperial College London, London, United Kingdom; 3 The DeWorm3 Project, The Natural History Museum of London, London, United Kingdom; New York Blood Center, UNITED STATES

## Abstract

**Background:**

The current World Health Organization (WHO) target for the three major soil-transmitted helminth (STH) infections is to reduce prevalence of moderate-to-heavy infections to below 1% by 2020. In terms of monitoring and evaluation (M&E), the current WHO guidelines for control of STHs recommend evaluation of infection levels in school-age children (SAC) after five to six years of preventive chemotherapy (PC), using the standard Kato-Katz faecal smear. Here, we assess the predictive performance of various sampling designs for the evaluation of the morbidity target.

**Methodology/Principal findings:**

Using two mathematical models for STH transmission and control, we simulate how the number of villages and SAC sampled affect the ability of survey results in sentinel villages to predict the achievement of the morbidity target in PC implementation units (e.g. districts). As PC is stopped when the prevalence of infection in SAC in sentinel villages is less than 1%, we estimate the positive predictive value (PPV) of this indicator for meeting the morbidity target in the whole district. The PPV varies by species and PC strategy, and it is generally higher in areas with lower pre-control prevalence. Sampling a fixed number of SAC spread out over 10 instead of 5 sentinel villages may increase the PPV by up to 20 percentage points. If every SAC in a village is tested, a higher number of villages may increase the PPV by up to 80 percentage points. Increasing the proportion of SAC tested per village does not result in a relevant increase of PPV.

**Conclusions/Significance:**

Although the WHO guidelines provide a combined strategy to control the three STH species, the efficacy of PC strategies clearly differs by species. There is added value in considering more villages within implementation units for M&E of morbidity targets, the extent varying by STH species. A better understanding of pre- and post-control local STH prevalence levels is essential for an adequate M&E strategy including the definition of morbidity targets at the appropriate geographical scale.

## Author summary

Soil-transmitted helminths (STHs) affect approximately 1.5 billion people worldwide. The World Health Organization target for STHs is to achieve <1% prevalence of moderate-to-heavy infections among school-age children (SAC) through preventive chemotherapy (PC) with albendazole or mebendazole. For monitoring and evaluation (M&E) of STH infections, it is recommended to assess the infection levels in SAC after five to six years of treatment and to stop PC if the prevalence of infection is <1%. In this study, we use two mathematical models to assess the predictive performance of different sampling designs for the evaluation of the morbidity target. We find that the efficacy of PC strategies differs significantly by species and pre-control infection levels. Achieving 1% prevalence of infection in sentinel villages may still imply a prevalence of moderate-to-heavy infections >1% in the entire PC implementation unit. Evaluating the prevalence of any infection over a higher number of sentinel villages improves the accuracy in assessing the morbidity target at the implementation unit level, even when a lower proportion of SAC per village is tested. A better understanding of pre- and post-control local STH prevalence levels through large scale data collection is essential for the definition of an adequate M&E strategy for STH control.

## Introduction

Approximately 1.5 billion people are infected with soil-transmitted helminths (STHs) worldwide [1]. The main STH species are *Ascaris lumbricoides* (roundworm), *Trichuris trichiura*, (whipworm) and hookworm (*Ancylostoma duodenale and Necator americanus*). Morbidity due to STHs is closely related to worm burden: chronic, moderate and high-intensity STH infections cause malnutrition, anaemia, stunted growth and impaired physical development in children [1]. The World Health Organization (WHO) global target for STHs is to eliminate morbidity in high-risk groups by 2020, defined as achieving less than 1% prevalence of moderate-to-heavy infections among pre-school-age children (preSAC, age 2–5), school-age children (SAC, age 5–14), and women of childbearing age [2]. Current WHO treatment guidelines recommend annual or semi-annual preventive chemotherapy (PC) using single-dose albendazole or mebendazole with a coverage of at least 75% of the aforementioned risk populations [1]. In practice, PC programmes are mostly school-based and therefore cover SAC and in some situations preSAC as well. PC is implemented semi-annually if the prevalence of any STH infection in SAC is higher than 50%, and annually if STH prevalence is between 20 and 50%. No PC is implemented if the pre-control STH prevalence is below 20%.

The WHO strategic plan for 2011–2020 [2] provides some recommendations on the monitoring and evaluation (M&E) of STH control programmes. However, they lack details on the recommended implementation of M&E and the interpretation of M&E results. Providing clear criteria to decide when to scale up/down or to stop PC would be very useful to guide programmes towards achieving the control goals [3]. Current recommendations [2] state that infection levels in SAC should be evaluated five to six years after starting annual or semi-annual PC. Based on the results, the decision is taken to either 1) continue PC as implemented initially (once or twice per year), 2) scale up PC frequency to three times per year, 3) scale down PC frequency to once every year or once every two years, or 4) stop PC altogether. The latter happens if the prevalence of infection (any intensity) in SAC is less than 1% [2]. All these criteria [4] are based on assessing the prevalence and intensity in sentinel sites selected proportionally to the number of SAC living in each ecological zone, using the Kato-Katz (KK) diagnostic method. Ecological zones are defined by the Food and Agriculture Organization of the United Nations (FAO) [5]. A clear indication on the number of sites and SAC to be sampled is lacking, as is the definition of standard indicators for M&E [2].

Previous work [6] has showed that there are practical difficulties in defining ecozones and in allocating different implementation units (e.g. districts consisting of a number of villages) to a single ecozone. Also, the impact of control programmes is likely non-homogeneous within ecological zones due to varying PC strategies in the different implementation units. In a comparison of survey designs for STH M&E in Kenya [6], using the recommended WHO approach (districts are aggregated according to ecological zone), appeared to be the least accurate sampling strategy.

A thorough understanding on how to assess the achievement of the prevalence thresholds to scale down or stop PC and how they relate to the achievement of the morbidity goal is currently lacking. In this study, we use two individual-based stochastic models developed independently by research groups at Imperial College London (ICL) and Erasmus Medical Center Rotterdam (Erasmus MC) to investigate how well different M&E strategies based on different sampling designs can detect whether the morbidity target of less than <1% prevalence of moderate-to-heavy intensity infections is achieved in a district after five to six years of PC.

Since the decision on whether to continue PC after the evaluation at five to six years is made by the programs at the level of PC implementation units, we construct ensembles of stochastic simulations that represent villages within districts, according to a realistic distribution of pre-control prevalence of infection. The models are then used to simulate the distribution of prevalence and intensity levels after five to six years of PC. Different post-control M&E strategies are then modelled in terms of different sampling schemes, i.e. different numbers of sentinel villages per district and different proportions of SAC sampled per sentinel village. Finally, we calculate the positive predictive value (PPV) of the indicators built using the prevalence of any infection in SAC (as measured by a single KK slide) and several threshold values to predict the achievement of <1% prevalence of moderate-to-heavy infection in all SAC in the entire district. When the threshold value is chosen to be 1%, this corresponds to assessing the probability that stopping PC in the implementation area correctly reflects the achievement of the morbidity target 5 years after the start of STH control.

## Methods

### General approach

The two models developed by Erasmus MC and ICL are individual-based stochastic transmission models, that allow the simulation of different STH transmission conditions and the impact of different control strategies. Both models are based on similar biological and demographic assumptions, the acquisition and death of worms are stochastic, with species-specific mean lifespans. In both models, exposure and contribution of the worms to the infective pool in the human habitat (i.e. practice of open defecation) are age-specific but differ in the functional forms and parametrisation. Based on the age pattern in hookworm infection levels, the Erasmus MC model assumes that the practice of defaecation increases with age up to age ten, and this pattern in open defaecation is then also applied to *T*. *trichiura* and *A*. *lumbricoides*. The ICL model assumes that age-dependent contribution is proportional to age-dependent exposure, and therefore it differs among the three species. Formal description of the Erasmus MC model has been published previously [7,8]. The individual-based ICL model has been presented in previous studies [9,10] and described in its deterministic version in earlier work [11]. Further details on specific assumptions, functional forms and parameter values can be found in S1 Table.

### District generation with predefined pre-control prevalence distribution

The simulation approach used in this work constructs ensembles of stochastic model realisations that represent villages within districts. The villages are independent units, and there is no exchange of individuals among villages. Each district is defined by a specific distribution of pre-control infection levels, characterised by a given mean and variance. To construct the districts, we first use the two mathematical models for the transmission of STHs developed by Erasmus MC and ICL to simulate a large pool of villages with stochastic transmission conditions, defined in terms of transmission rate for Erasmus MC model and basic reproduction number (i.e. R_0_, indicating the transmission intensity in a defined setting) for the ICL model. In both, the level of exposure heterogeneity is maintained fixed (values can be found in S1 Table). For each village, we simulate a pre-control prevalence of infection in SAC at baseline, measured using a single KK slide taken from all SAC in sentinel villages before the start of PC. Then, we assign a normalising weight to each village, based on the inverse of the density of its pre-control prevalence within the larger pool of village simulations, using a Gaussian kernel. The weights are used to repeatedly generate districts of 150 villages with a given desired distribution of pre-control prevalence of infection. Each village consists of approximately 500 simulated individuals. The choice of the population size and number of villages by district was informed by high resolution population count data generated within the WorldPop project [12,13] and by the implementation units shapefile for Sub-Saharan Africa available as part of the interactive mapping tool for control NTDmap [14].

We assume that the distribution of pre-control prevalences in a district follows a beta distribution with mean *μ* in the range between 0.2 and 0.4 (with 0.01 increments). To have these beta distributions represent a realistic level of geographical variation within a district at pre-control, we use sub-Saharan pixel-level prevalence predictions published in 2014 [15]. We aggregated these predictions over implementation units, and both mean and variance of implementation units were weighted by pixel-level population densities. Then a linear model was fitted to the log-transformed variance *σ*^2^ of the distribution of pre-control prevalences and its logit-transformed mean *μ*:

$$\log\left(\sigma^{2}\right)=\tau_{species}+\theta_{species}logit(\mu )$$ (1)

where *τ*_species_ and *θ*_species_ indicate the species-specific intercept and slope, respectively. The values for each species used here are reported in Table 1. Based on the available data, our analysis showed that for hookworm and *T*. *trichiura* the prevalence variation within a district increases as the mean prevalence *μ* increases. In contrast, the prevalence variation for *A*. *lumbricoides* does not depend on the mean prevalence and remains constant. Mean and variance were then used to obtain the shape parameters of a corresponding beta distribution employed for simulating implementation units.

**Table 1:** Coefficients for the linear association between the mean and standard deviation in prevalence of STH infection within districts. These parameters are meant to represent geographical variation in STH prevalence at the level of implementation units.

Species	Intercept	Slope
**Hookworm**	-7.50	1.27
***A*. *lumbricoides***	-8.30	0.00
*T*. *trichiura*	-8.82	0.23

### PC simulation

PC strategy is decided at the district level: annual or semi-annual PC, targeting preSAC and SAC (ages 2–15) through schools, or treating the whole community (age 2 and above). For each village in a district we simulate the dynamics of STH infection levels using four treatment strategies (school-based annual PC, school-based semi-annual PC, community-wide annual PC and community-wide semi-annual PC). We only model treatment with albendazole, which is assumed to kill parasites with a probability of 99% (*A*. *lumbricoides*), 94% (hookworm), or 60% (*T*. *trichiura*), in line with measured egg reduction rates [16,17]. We assume a mean treatment coverage of 75% of the target population, with 95% of the villages having coverage between about 65% and 85%. This corresponds to a beta distribution with shape parameters *α* = 52.5 and *β* = 17.5 (mean 0.75, 95% CI (0.643–0.843)).

### Sampling strategies

**Fig 1:**
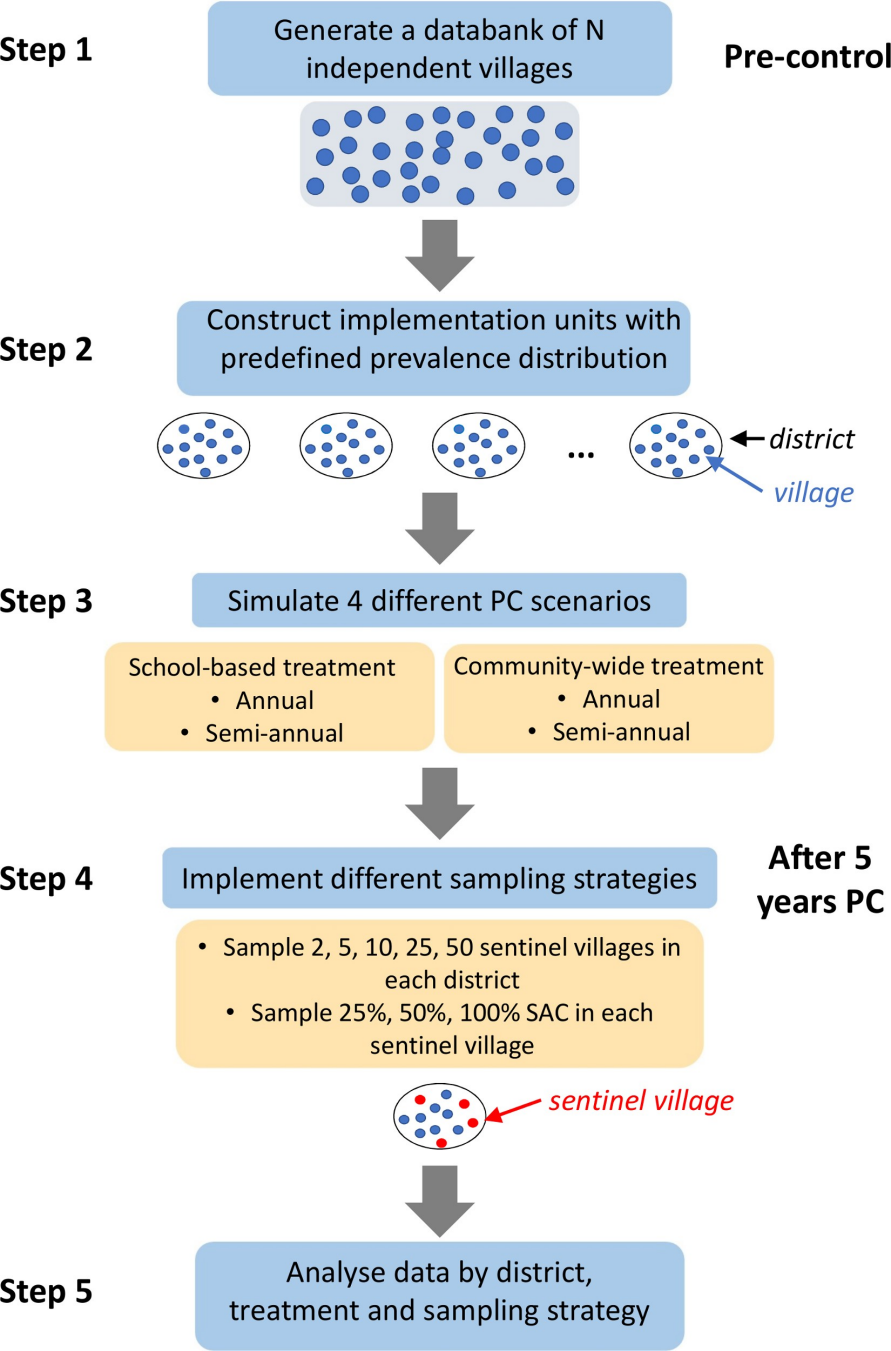
Flowchart describing the different steps of the simulation approach. First, each of the two models was used to generate a databank of simulations (step 1). Each simulation with a (closed) population size of about 500 individuals can realistically represent transmission within a village. In step 2, implementation units are simulated sampling villages according to a predefined prevalence distribution according to Eq (1). The models are then used to simulate the impact of 4 different PC strategies on prevalence and intensity of infection (step 3). In step 4 (post-control) we consider 4 different sampling strategies and in step 5 we analyse the results by district, PC and sampling strategy.

The flowchart in Fig 1 summarises all the different steps of the simulation methods. Further details on the sampling methodology, assumed drug efficacy and other parameters are described in S1 Table. For each generated district, we randomly select a sample of N sentinel villages (N = 2, 5, 10, 25, or 50) and evaluate the baseline prevalence in 100%, 50% or 25% of SAC in these villages before start of PC, using a single-slide KK per person. Five years after the start of PC, simulated single-slide KK measurements are taken again from a new sample of SAC to evaluate prevalence in the same sentinel villages. We extract model predictions for prevalence of infection in SAC in sentinel villages (the indicator) and the prevalence of moderate-to-heavy intensity infection in SAC in all villages as measured by a single-slide KK (the “true” outcome). We then calculate the PPV of the sentinel-village-level indicator for the district-level “true” outcome as the ratio A / B. The denominator B is the number of simulated districts with sentinel-village-level prevalences under a given threshold, and the numerator A is the number of simulated districts among B where the “true” prevalence of moderate-to-heavy infection is below 1% (i.e. achievement of the morbidity target). When the threshold value is chosen to be 1% and the predictor is the prevalence of any infection in the sentinel villages, the PPV corresponds to the proportion of districts interrupting PC, that have in fact reached the morbidity target 5 years after the start of STH control.

## Results

### Prevalences of infection in SAC at the district level

**Fig 2:**
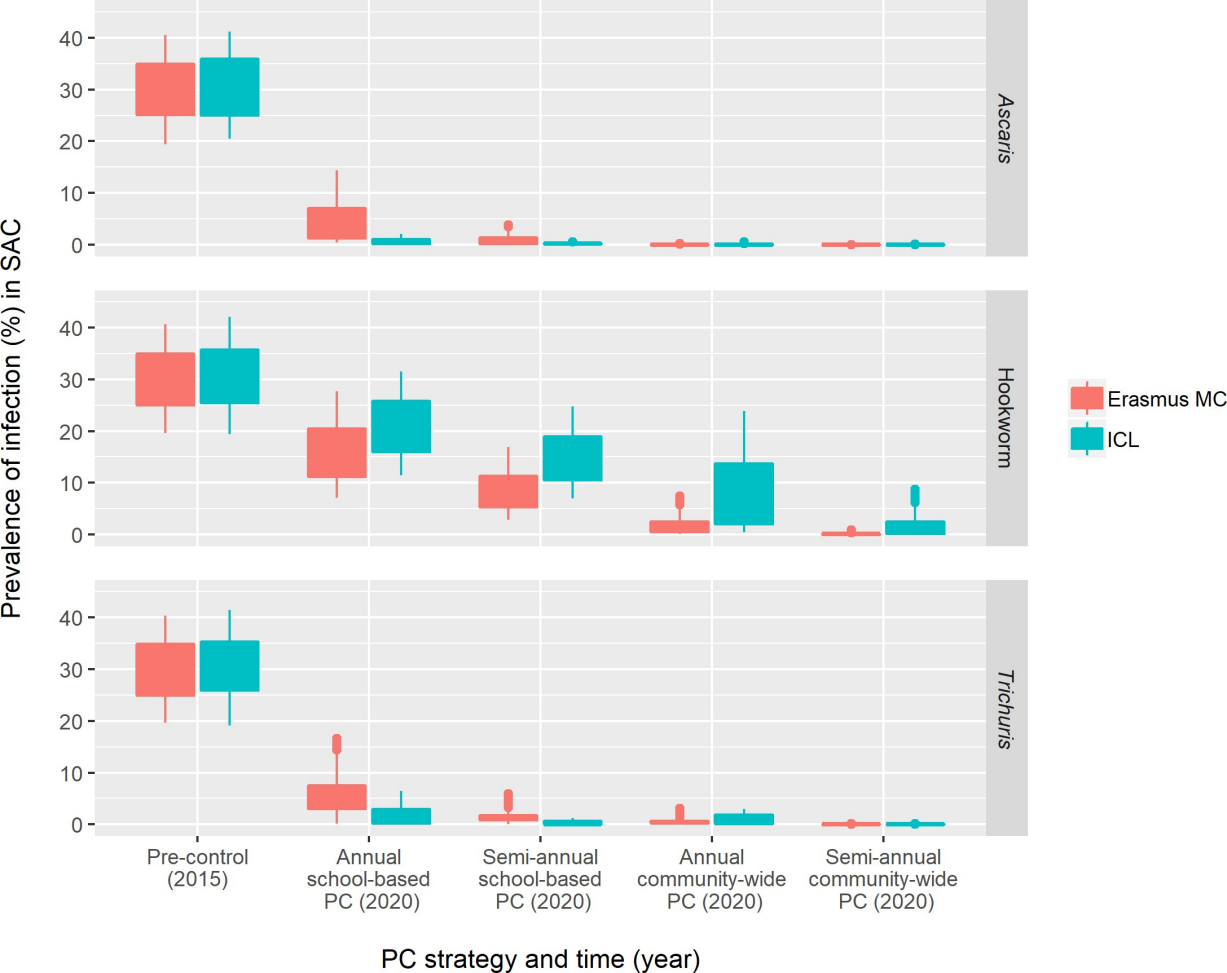
Box-plots of model-predicted prevalences of infection among school-age children (SAC) at the district level before and after school-based PC. Prevalence is measured in all SAC living in each district at two time points: at baseline (2015) and after 5 years of PC (2020). Every randomly generated district has mean baseline prevalence between 20 and 40% (0.01 increments). School-based PC is assumed to cover children of age 2–15 (preSAC and SAC) at 75% and community-based PC is assumed to cover the entire population of age >2 at 75% (allowing for random variation in coverage between individual villages within the district). S1 Fig holds similar plots for results stratified by mean baseline prevalence in districts (20–30% and 30–40%).

The distribution of pre- and post-control prevalences of STH infection in SAC in simulated districts is displayed in Fig 2, with prevalences based on KK testing of all SAC in the districts before the start of PC (2015) and after 5 years of PC (2020). Both Erasmus MC and ICL models adequately produce the predefined distribution of district level prevalences at baseline.

The two models qualitatively agree on the impact of PC on STH prevalence levels on any infection in SAC: community-wide PC vs. school-based PC and semi-annual vs. annual PC more strongly reduce the prevalence of any infection in SAC for all species. Because in the ICL models for *T*. *trichiura* and *A*. *lumbricoides*, adult humans are assumed to practice open defecation less frequently as they get older (i.e. age-dependent contribution to transmission is proportional to age-dependent exposure to transmission), school-based PC has a higher impact than predicted by the Erasmus MC model for these two species. Conversely, because in the Erasmus MC open defecation practices are assumed to be stable after the age of ten, adults contribute more to *T*. *trichiura* and *A*. *lumbricoides* transmission than in the ICL model, and hence, there is a larger additional benefit of implementing community-wide PC than predicted by the ICL model. S1 Fig compares baseline and post-control prevalences between the two models for district level means between 20 and 30% (first page) and between 30 and 40% (second page).

### District-level achievement of the morbidity target after 5 years of PC

**Fig 3:**
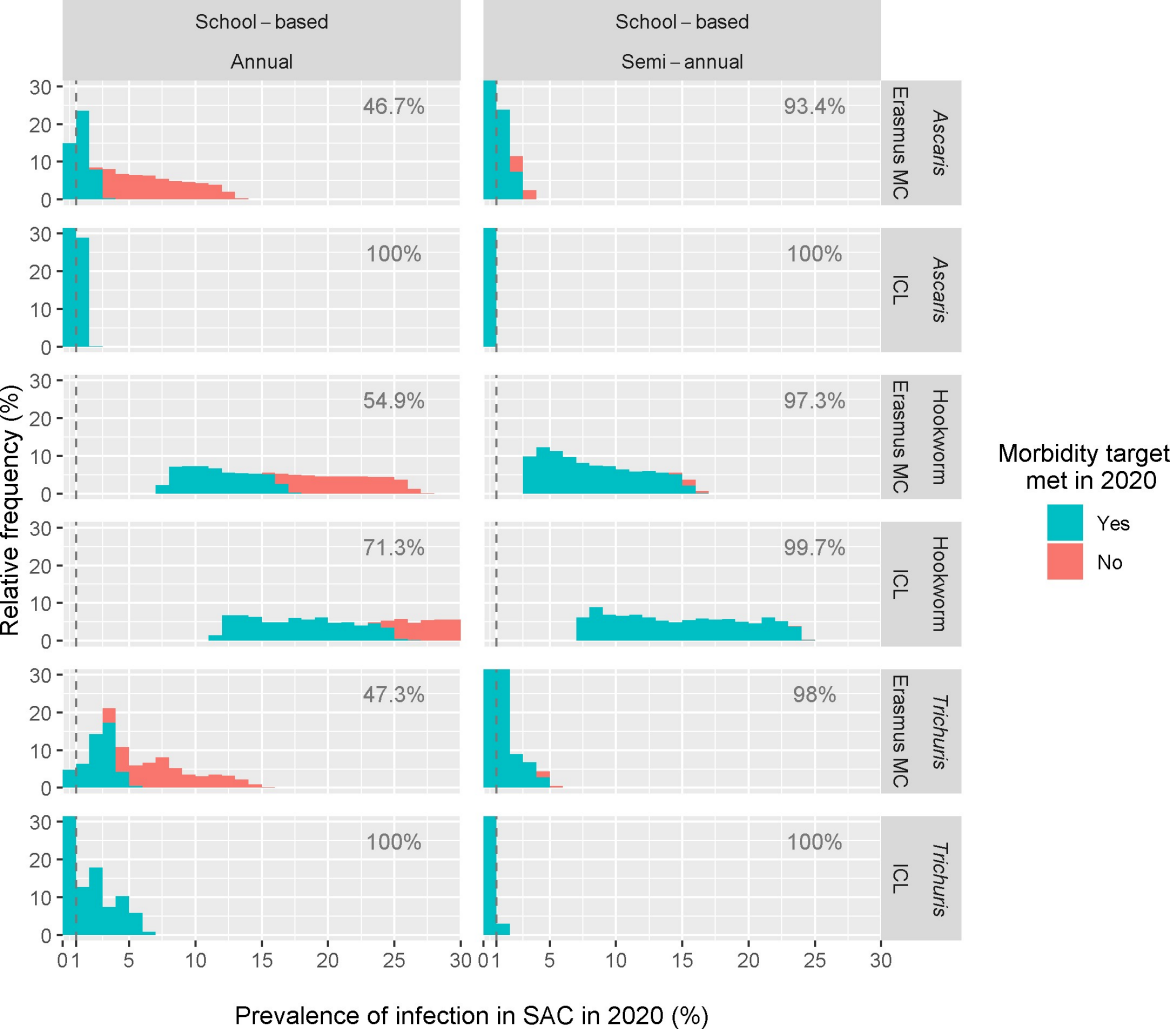
Model-predicted distribution of prevalence of STH infection at the district level in SAC in 2020. The stacked histogram is used to distinguish between districts that have met the morbidity target in 2020 (turquoise) and those that have not (red). Meeting the morbidity target refers to reaching <1% moderate-to-heavy infections in SAC. The dashed line at 1% represents the recommended prevalence threshold of any infection among SAC required to stop PC. The numbers in grey in each panel represent the overall probability of meeting the target (i.e. the proportion in turquoise). Prevalence is assumed to be measured in all SAC living in each randomly generated district with mean baseline prevalence ranging from 20 to 40%. Only school-based PC are shown as community-based PC always resulted in meeting the morbidity target. S2 Fig provides additional plots stratified by the average pre-control prevalence in a district (20%-30% vs. 30%-40%).

The histograms in Fig 3 show the post-control prevalence distribution of any (i.e. low, moderate and/or heavy) STH infection in SAC and the threshold value of 1% (vertical dashed line) used to make the decision of stopping PC. The plot further distinguishes between simulated districts that meet the morbidity target (prevalence of moderate-to-heavy infections <1%, turquoise) and those which do not meet the morbidity target (red). The bars to the left of the dashed lines (prevalence of any infection <1%) are entirely turquoise (as expected) because the morbidity target is always met in those districts. Considerable differences in these distributions are detectable depending on the PC target population, PC frequency, and the STH species (see S2 Fig for comparison stratified by baseline prevalences of 20–30%, first page, and 30–40%, second page).

Across all species, both models suggest that a school-based semi-annual PC is almost always a successful strategy for reaching the STH morbidity target (Fig 3) for the considered baseline prevalence levels. Community-wide PCs (both annual and semi-annual) resulted in the achievement of the morbidity target at district level for both mean district prevalence levels between 20 and 30% (S2 Fig, first page) and mean district prevalence levels between 30 and 40% (S2 Fig, second page).

According to our simulations, for hookworm intensive PC (school-based semi-annual PC and both the community-wide PC strategies) almost always meet the morbidity target in 2020, even if the prevalence of any infection in SAC is considerably higher than 1%. Five years of annual school-based PC is only sometimes sufficient to reach the morbidity target (54.8% vs. 71.3%, Erasmus MC and ICL model, respectively).

Furthermore, depending on STH species and PC strategy, the feasibility of reaching the morbidity target also depends on the pre-control prevalence levels (S2 Fig). Districts with higher endemicity (30–40%) have a lower chance to achieve a prevalence below 1% of moderate-to-heavy infections.

### Predictive value of prevalence of infection in sentinel villages

The prevalence of STH infection in sentinel villages after 5 to 6 years of PC as assessed by a single slide KK is used to determine whether PC should be scaled up/down or stopped altogether in an implementation unit. Table 2 shows the probabilities of scaling down or stopping PC prematurely based on two different sampling strategies: 2 sentinel villages per implementation unit where only 25% of SAC is tested for STH infection versus 50 sentinel villages with all SAC tested. Sampling more villages (and more SAC per village) reduces considerably the misclassification probabilities for treatment allocation at the district level.

**Table 2:** Model-based misclassification probabilities for PC allocation at district level after 5 years of PC. For each sampling strategy (2 villages, 25%SAC vs 50 villages, 100% SAC) the probability of scaling down or stopping PC is reported against the treatment strategy that is required (based on the true prevalence at district level). The first line represents the probability as assessed by repeated runs using Erasmus MC model and the second row using ICL model.

		Treatment strategy as evaluated by sentinel villages					
		2 villages, 25% SAC			50 villages, 100% SAC		
**WHO treatment strategy in the district (required)**		**Stop PC**	**PC once/2 years**	**PC once/year**	**Stop PC**	**PC once/2 years**	**PC once/year**
	**PC once/2 years**	30.86%			4.18%		
		30.86%			5.31%		
	**PC once/year**	0.70%	27.23%		0%	3.51%	
		0%	19.05%		0%	1.02%	
	**PC twice/year**	0%	2.10%	26.05%	0%	0%	4.13%
		0%	0.79%	23.84%	0%	0%	3.51%

**Fig 4:**
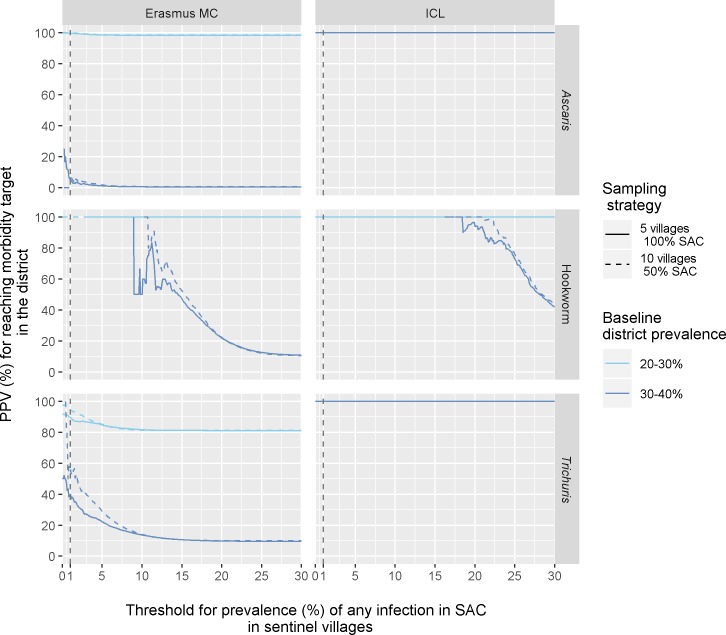
Positive predictive value of prevalence of STH infection (any intensity) in SAC in sentinel villages for meeting the morbidity target in 2020 at the district level. The y-axis represents the probability that the morbidity target is met in 2020 (prevalence of moderate-to-heavy infections in SAC <1% in the district). The x-axis represents the threshold value for prevalence of any infection in SAC in sentinel villages, as measured with a single-slide Kato-Katz. Line colours indicate results stratified by mean district baseline prevalence of infection in SAC. The line type indicates different sampling strategies but with the same total number of SAC tested. Only school-based annual PC is shown as more intensive PC always resulted in meeting the morbidity target.

Fig 4 shows the PPV for reaching the morbidity target for STH infection in a district given potential threshold values for the prevalence of infection (any intensity) in SAC in sentinel villages, comparing sampling of all SAC in 5 sentinel villages (solid lines) with sampling 50% of SAC in 10 sentinel villages (dashed lines). Only school-based annual PC is considered as the morbidity target was always met under more intensive PC strategies. In general, the PPV increases with lower threshold values, unless the morbidity target is never or always met in 2020, regardless of the prevalence of infection in sentinel villages (i.e. horizontal lines at either the bottom or top of the graph, respectively). Further, the PPV curve for districts with average pre-control prevalences in the range 20–30% (light blue) lies above the PPV curve for prevalence values between 30 and 40% (dark blue). This means that for districts with a lower pre-control prevalence (20–30%) higher threshold values can be used as an indicator for having met the morbidity target in 2020. In general, for a given pre-control prevalence level, the PPV curve associated with testing all SAC in 5 sentinel villages and PPV curve describing the sampling of 50% SAC in 10 sentinel villages coincide, suggesting that little additional information is provided by sampling the same total number of children from more villages. However, for threshold values close to 1%, the PPV curve based on sampling 10 villages and 50% SAC lies up to 20 percentage points higher than the curve for testing all SAC in 5 sentinel villages for high baseline prevalences (30–40%).

Using the prevalence of moderate-to-heavy infection measured in the sentinel villages as an indicator of meeting the morbidity target at district level (S3 Fig) shows a lower PPV but allows a smaller and consistent threshold across the three STH species.

**Fig 5:**
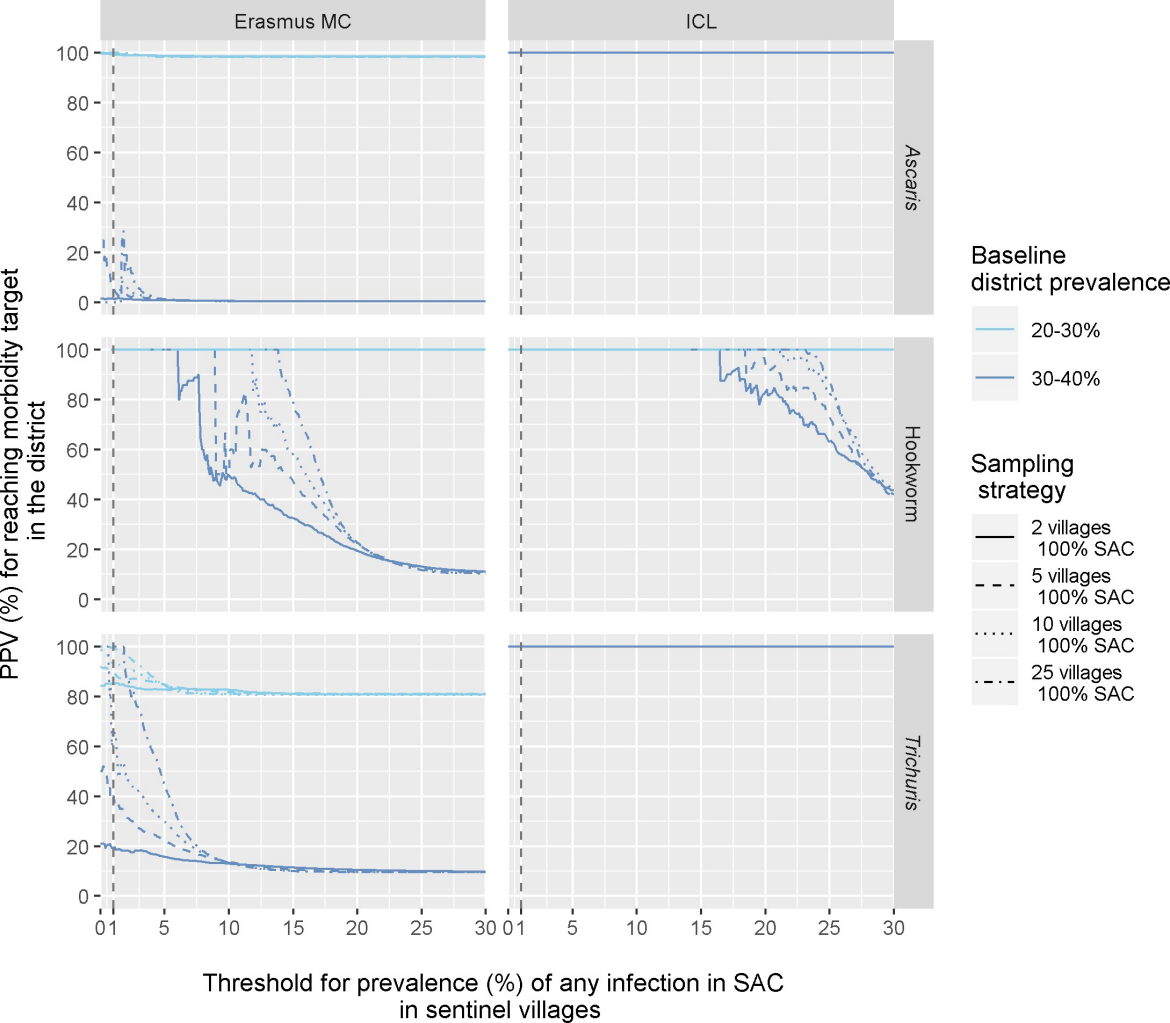
Impact of number of sampled sentinel villages on predictive value of prevalence of any infection in SAC for meeting the morbidity target in 2020 at the district level. The y-axis represents the probability that the morbidity target is met in 2020 (prevalence of moderate-to-heavy infections in SAC <1%, averaged over all villages in the district). The x-axis represents the threshold value for prevalence of any infection in SAC in sentinel villages, as measured with a single-slide Kato-Katz. Line colours indicate results stratified by mean district baseline prevalence of infection in SAC. The line type indicates different number of villages sampled maintaining the same proportion of SAC (100%). Only school-based annual PC is shown as more intensive PC always resulted in meeting the morbidity target.

Comparing sampling strategies that considers an increasing number of villages (2, 5, 10, 25 per implementation unit) with a fixed proportion of SAC tested per village (Fig 5) confirms the added value in terms of PPV, with a difference of up to 80 percentage points between the two extreme cases (2 villages vs. 25 villages testing all SAC). On the other hand, increasing the total population sampled by testing more SAC per village (S4 Fig) leads to a limited increase of the PPV for meeting the morbidity target.

## Discussion

We have investigated the performance of different M&E strategies in assessing whether the STH morbidity target is reached by 2020, using two stochastic individual-based transmission models developed by researchers at Erasmus MC and Imperial College London. Model predictions are in good agreement. In general, we found that the feasibility of meeting the morbidity target varies by species and baseline prevalence. For the range of prevalences considered here (20–40%) school-based PC with an annual frequency is not always sufficient to achieve <1% prevalence of moderate-to-heavy infections in SAC. Sampling strategies that involve few sentinel villages per implementation unit pose a considerable risk of prematurely scaling down/stopping PC. The positive predictive value (PPV) of prevalence of infection in SAC in sentinel villages for meeting the WHO morbidity target in a district is generally higher for areas with lower pre-control prevalence levels (lower *R*_0_ values) and/or past higher-intensity PC strategies. Sampling a fixed number of children spread out over 10 instead of 5 sentinel villages may increase the PPV by up to 20 percentage points. If the fraction of SAC sampled is fixed (e.g. 100%) increasing the number of sampled villages from 2 to 25 may result in a PPV up to 80 percentage points higher. On the other hand, for a random sample of 5 sentinel villages, testing of 100% instead of 50% or 25% of the SAC in those villages provides very little additional information. Furthermore, given that PC against STH infections is currently typically school-based, the WHO-recommended criterion of 1% prevalence of any infection in SAC as a marker of success is generally too optimistic for areas that have received 5 years of PC. The PPV for achieving the morbidity target is often well under 90% or even zero, except for the very low-endemic areas (low *R*_0_ values for the ICL model and low transmission rate for Erasmus MC) where the morbidity target is always met, regardless of the prevalence of any infection in SAC.

One of the novelties of this work is that we used the models to construct ensembles of stochastic simulations that represent villages within districts according to a defined distribution of pre-control prevalences of infection. The probability distributions of prevalence values represent local geographical variation in infection levels across villages in the same district. This description is important both because it is what is observed in large spatially structured epidemiological studies of STH infection in endemic regions and because the decision of whether to continue PC after the evaluation at 5 years is made by the programmes at the district level. In our analysis we based our assumptions about geographical variation within districts on an analysis of previous work by Pullan and colleagues [15] where a geostatistical model was used to obtain pixel-level estimates of STH prevalence in sub-Saharan Africa using all collated survey data until 2010. However, variation of prevalence of STH infection in specific localities at finer spatial scale may be different from what we assume here. Therefore, to produce model predictions for more specific situations it is important to gather more data and/or to collate existing data on the geographical variation in pre-control STH prevalences at clearly defined spatial scales. Two major Bill and Melinda Gates Foundation funded studies (the Tumikia study in Kenya [18] and the DeWorm3 study in India, Benin and Malawi [19]) will provide very detailed information on the heterogeneity in STH prevalence in large populations at village-level spatial scale in the near future. Given such information and the history of PC in an area, along with information or assumptions about the variation in local PC coverage, the simulations methods described in the paper can provide insight into the appropriateness of different sampling schemes for specific areas. For instance, for districts with higher variation in village-level prevalence of infection than in our analysis (e.g. because of higher variation in pre-control transmission conditions and/or patchy geographical coverage of PC), the benefit of sampling of additional sentinel villages would be higher than we predict here. A better understanding of the pre- and post-control variation in local STH prevalences will be essential to improve the definition of the WHO morbidity target and to define the appropriate geographical scale to consider in monitoring and evaluation progress towards the defined targets.

A current limitation of our approach is that we consider villages within a district as isolated units, such that no infections can be transferred between villages within a geographical area. Also, in our analysis, we assumed a roughly stable PC coverage (geographically), meaning that infection levels in all villages within the simulated district decline synchronously. This means that our model predictions are relatively optimistic particularly for areas where the geographical coverage of PC within an implementation unit is irregular such that spill-over of infection from untreated to treated villages becomes relevant, especially close to elimination. Such areas require more detailed and sophisticated modelling of human movement patterns to account for spill-over of infection between villages, which theoretically has a stabilising effect on infection levels and impedes achievement of control or elimination. Work is in progress on developing both stochastic frameworks of movement patterns and analytical studies of how such movements influence breakpoints in transmission.

Overall, we found that achieving the morbidity target is highly dependent on the dominant STH species, PC strategy and pre-control prevalence levels (transmission intensities in defined localities). The WHO treatment guidelines provide a combined strategy to control the three STH species. However, differences are evident in their responses to the different PC strategies and therefore also in the required action to meet the WHO morbidity goal. We would therefore advocate for an approach that differentiates among the three main species, such that the implemented policy is based on whatever dominant species present requires in terms of intervention and the associated M&E strategy. Within the current scope of WHO-recommended strategies (i.e. school-based deworming of SAC and preSAC) this means that five years of PC is too short a period to start considering evaluation of the morbidity target. To achieve the morbidity target within five years, community-wide treatment would need to be considered, and/or treatment with a combination of albendazole and ivermectin (not modelled here), which is more effective against *T*. *trichiura* in particular [20,21].

As previously pointed out by the STH Advisory Committee [22], survey methods currently endorsed by WHO to assess the prevalence of any STH infection are not designed to determine whether or not the morbidity goal in children has been achieved. Therefore, it may be advisable to evaluate achievement of the morbidity target using the prevalence of any infection in combination with the estimated proportion of moderate to heavy infections in the sentinel villages. The current recommended diagnostic is egg count through means of a KK, and therefore a direct quantification of moderate and heavy infection is possible. There would still be uncertainty due to the limited number of villages and people tested but the assessment of the real morbidity level in the district could be substantially improved and the definition of a threshold value to stop PC would be more straightforward. However, in terms of PPV, the use of prevalence of any infection in SAC as a criterion to stop PC remains the safest (S4 Fig).

The potential added value of more sensitive diagnostics such as multiple slide KK or quantitative (real-time) polymerase chain reaction (qPCR) is object of current investigation and will provide useful information for new WHO guidelines for the 2021–2030 period, also in terms of M&E sampling strategies to assess the morbidity target.

The current WHO treatment guidelines provide a combined strategy to control the three STH species. However, the efficacy of PC strategies clearly differs by species given differences in both drug efficacy and the age distribution of infection, with each having a different optimal strategy to meet the morbidity target. Meeting the criterion of 1% prevalence of any infection in district sentinel villages may still mean that the prevalence of moderate-to-heavy infections is higher than 1% in a considerable part of the district. Large-scale data collection through well designed M&E programmes that include information on mass drug administration coverage and individual longitudinal compliance to treatment, combined with further simulation studies, are required to further our understanding of how best to achieve the WHO STH control goals and how best to monitor and evaluate progress.

## Supporting information

S1 Table Model parameters used to simulate transmission of *Ascaris lumbricoides*, *Trichuris trichiura* and hookworm infections. (DOCX)

S1 Fig Box-plots of model-predicted prevalences of infection among school-age children (SAC) at the district level before and after different PC strategies. Prevalence is measured in all SAC living in each district at two time points: at baseline (2015) and after 5 years of PC (2020). Every randomly generated district has mean baseline prevalence between 20 and 40% (0.01 increments) (first page). School-based PC is assumed to cover children of age 2–15 (preSAC and SAC) at 75% and community-based PC is assumed to cover the entire population of age >2 at 75% (allowing for random variation in coverage between individual villages within the district). Every randomly generated district has mean baseline prevalence between 20 and 30% (second page) or 30% and 40% (third page).
(PDF)

S2 Fig Model-predicted distribution of prevalence of infection at the district level in SAC in 2020. The stacked histogram is used to distinguish between districts who have met the morbidity target in 2020 (*turquoise*) and those who have not (*red*). Meeting the morbidity target refers to reaching <1% moderate to heavy infections in all SAC in all villages (as measured by a single-slide Kato Katz). The dashed line at 1% represents the recommended threshold in prevalence of any infection among SAC required to stop PC. The numbers in grey in each panel represent the overall probability of meeting the target (i.e. the proportion of the histogram that is turquoise). Prevalence is assumed to be measured in all SAC living in randomly generated district with mean baseline prevalence ranging from 20% to 40% (first page), 20% to 30% (second page), or 30% to 40% (third page).
(PDF)

S3 Fig Positive predictive value of prevalence of STH infection (any intensity vs moderate-to-heavy intensity) in SAC in sentinel villages for meeting the morbidity target in 2020 at the district level. The y-axis represents the probability that the morbidity target is met in 2020 (prevalence of moderate to heavy infections in SAC <1% in the district). The x-axis represents the threshold value for prevalence of any infection or prevalence of moderate-to-heavy infections in SAC in sentinel villages, as measured with a single-slide Kato Katz. Line colours indicate results stratified by mean district baseline prevalence of infection in SAC. The line type indicates the different prevalence indicator. Only school-based annual PC is shown as more intensive PC always resulted in meeting the morbidity target.
(PDF)

S4 Fig Impact of proportion of children sampled per sentinel village on predictive value of prevalence of any infection in SAC for meeting the morbidity target in 2020 at the district level. The y-axis represents the probability that the morbidity target is met in 2020 (prevalence of moderate to heavy infections in SAC <1%, averaged over all villages in the district). The x-axis represents the threshold value for prevalence of any infection in SAC in sentinel villages, as measured with a single-slide Kato Katz. Line colours indicate results stratified by mean district baseline prevalence of infection in SAC. The line type indicates different proportion of SAC sampled per village, maintaining the total number of sentinel villages constant (5). Only school-based annual PC is shown as more intensive PC always resulted in meeting the morbidity target.
(PDF)
