# Supplementary material for: Sampling strategies for monitoring and evaluation of morbidity targets for soil-transmitted helminths
Source: PLoS Negl Trop Dis. 2019 Jun 26;13(6):e0007514. doi: 10.1371/journal.pntd.0007514 (PMC6615707; doi:10.1371/journal.pntd.0007514)
Supplement: S1 Table — (DOCX) [file pntd.0007514.s001.docx]

S1 Table. Model parameters used to simulate transmission of *Ascaris lumbricoides*, *Trichuris trichiura* and hookworm infections.

|  | **Value or assumption** | |
| --- | --- | --- |
| **Parameter** | **Erasmus MC** | **Imperial College London** |
| **Human demography** | | |
| *Hookworm* | Demographic data quantified for sub-Saharan Africa 2000 United Nations Population Division [1] | Demographic data taken from 2003 Kenya Demographic and Health Surveys [2]. |
| *Ascariasis* | Indian fertility and mortality rates as reported for 1980-1985 by United Nations Population Division (2015 Revision). | Demographic data taken from 2003 Kenya Demographic and Health Surveys [2]. |
| *Trichuris* | Indian fertility and mortality rates as reported for 1980-1985 by United Nations Population Division (2015 Revision). | Demographic data taken from 2003 Kenya Demographic and Health Surveys [2]. |
| **Transmission of infection** | | |
| Seasonal variation in contribution to reservoir | Stable throughout the year (assumption). | Stable throughout the year (assumption). |
| Aggregation of parasites in hosts |  |  |
| *Hookworm* | $k_{w}=0.35$ [2]. | $k_{w}=0.35$ [2]. |
| *Ascariasis* | $k_{w}=0.8$ [3]. | $k_{w}=0.8$ [3,4]. |
| *Trichuris* | $k_{w}=0.38$, fitted to data from [5]. | $k_{w}=0.38$, fitted to data from [5]. |
| Variation in exposure and contribution to the environmental reservoir by age and sex |  |  |
| *Hookworm* | Relative exposure and contribution to the reservoir both increase linearly from 0 to 1 between ages 0–10 and is stable thereafter with no difference between males and females [6]. | Relative exposure and contribution to the reservoir are assumed to vary piece-wise constant by age group, and are estimated at 0.12 (ages 0-15), 1 (ages 15-25), and 0.067 (ages 25+), assuming no difference between males and females. These figures were estimated from unpublished epidemiological data from Tamil Nadu. |
| *Ascariasis* | Contribution to the reservoir increases linearly from 0 to 1 between ages 0–10 and is stable thereafter with no difference between males and females (reflecting behaviour related to defaecation and mobility patterns as previously estimated for hookworm [6]). Exposure to the reservoir is defined as a piece-wise linear function of age that increases linearly from a base level $x_{0}$ =0.33 of relative exposure at age zero to a relative exposure of 1.0 at age $a_{\text{peak}}$=3, and then again linearly declines back to the base level $x_{0}$ at age 15 and is stable thereafter. This function aims to reflect behaviour leading to ingestion of contaminated matter, which typically peaks in young children [3]. | Relative exposure and contribution to the reservoir by age are assumed to be equal and are estimated from the baseline data: 0.22 (0-4 years), 1.88 (5-9), 1.0 (10-19), 0.53 (20+). |
| *Trichuris* | Contribution to the reservoir increases linearly from 0 to 1 between ages 0–10 and is stable thereafter with no difference between males and females (reflecting behaviour related to defaecation and mobility patterns as previously estimated for hookworm [6]). Exposure to the reservoir is defined as a piece-wise linear function of age that increases linearly from a base level $x_{0}$ =0.33 of relative exposure at age zero to a relative exposure of 1.0 at age $a_{\text{peak}}$=3, and then again linearly declines back to the base level $x_{0}$ at age 15 and is stable thereafter. This function aims to reflect behaviour leading to ingestion of contaminated matter, which typically peaks in young children [3]. | Relative exposure and contribution to the reservoir are assumed to vary piece-wise constant by age group and are estimated at 0.3 (0-4 years), 1.28 (5-14), 1 (15-24) and 0.17 (ages 25+), assuming no difference between males and females. These figures were estimated from epidemiological data from [5]. |
| **Life history and productivity of the parasite in the human host** | | |
| Average worm lifespan |  |  |
| *Hookworm* | 3 years [7–9]. | 2 years [10]. |
| *Ascariasis* | 1 year [3,7–9,11]. | 1 year [7–9,11]. |
| *Trichuris* | 1 year [5,10] | 1 year [5,10] |
| Variation in worm lifespan | Weibull distribution with shape 2; i.e. the mortality rate is zero at age zero and then increases linearly with worm age (assumption as previously used for hookworm [6]). | Exponential distribution; i.e. the mortality rate is constant and independent of worm age. |
| Pre-patent period |  |  |
| *Hookworm* | 7 weeks [7,8,12,13]. | No pre-patent period used. |
| *Ascariasis* | 10 weeks [7]. | No pre-patent period used. |
| *Trichuris* | 10 weeks [7]. | No pre-patent period used. |
| Age-dependent reproductive capacity | Constant over age (assumption). | Constant over age (assumption). |
| Female worm fecundity | Density-dependent on total number of female worms in host, assuming hyperbolic saturation [6]. | Density-dependent on total number of female worms in host, assuming exponential saturation. Exponential model of saturation with parameter γ = 0.02 [14] for hookworm, γ = 0.07 for ascaris [4] and γ = 0.0035 [5,15]. |
| *Hookworm* | On average 8.3 eggs per female worm per 41.7 mg sample of faeces (200 epg per female worm, as previously reported based on association between number of expulsed adult female worms and egg counts based on Kato-Katz [16]). The average maximum total host output is assumed to be 62.5 eggs per 41.7 mg faeces (1500 epg, as previously assumed [6]). | On average 3 eggs per female worm per 41.7 mg sample of faeces (72 epg per female worm, as previously reported based on association between number of expulsed adult female worms and egg counts based on Kato-Katz [16]). |
| *Ascariasis* | On average 406 eggs per female worm per 41.7 mg sample of faeces (9750 epg per female worm), and maximum total host output of 777 eggs per 41.7 mg faeces on average (18,650 epg). These figures were estimated from pre-control data on number of expulsed adult female worms and egg counts based on a concentration and sedimentation technique using homogenised stools [3]. | On average 320 eggs per female worm per 41.7 mg sample of faeces (7674 epg per female worm). |
| *Trichuris* | On average 15.4 eggs per female worm per 41.7 mg sample of faeces (370 epg per female worm), and maximum total host output of 3333.33 eggs per 41.7 mg faeces on average (80,000 epg). These figures were estimated from pre-control data on number of expulsed adult female worms and egg counts based on a concentration and sedimentation technique using homogenised stools [3]. | On average 5.875 eggs per female worm per 41.7 mg sample of faeces (141 epg per female worm) [5]. |
| Host immunity to incoming infections | None (assumption). | None (assumption). |
| **Infection dynamics in environmental reservoir** | | |
| Survival of infective material in the central reservoir | Exponential survival (assumption). | Exponential survival (assumption). |
| *Hookworm* | Average lifespan of two weeks, implemented as a monthly survival probability of $\exp\left( -26/12 \right)=11.5\%$ (95%-CI: 0.05–7.38 weeks under assumption of exponential survival), based on the notion that average survival time is in the order of weeks [12,13,17]. | Average lifespan of 30 days [10]. |
| *Ascariasis* | Average lifespan of 1.5 month, implemented as a monthly survival probability of $\exp\left( -1/1.5 \right)=51.3\%$ (95%-CI: 0.04–5.53 months under assumption of exponential survival) [8,9]. | Lifespan of approximately 2 months [18]. |
| *Trichuris* | Average lifespan of 20 days implemented as a monthly survival probability of $\exp\left( -1/(2/ 3 \right))=22.3\%$ (95%-CI: 0.02–2.46 months under assumption of exponential survival. | Lifespan of approximately 20 days [5]. |
| **Drug treatment** | | |
| Proportion of adult worms killed by single dose of albendazole (400 mg), or pyrantel pamoate (10 mg/kg, ascariasis only) | Assumption: proportion killed is equal to the faecal egg reduction rate. | Assumption: proportion killed is equal to the faecal egg reduction rate. |
| *Hookworm* | 0.95 for albendazole [19]. | 0.95 for albendazole [19]. |
| *Ascariasis* | 0.99 for albendazole [19] | 0.99 for albendazole[19]. |
| *Trichuris* | 0.60 for albendazole [19] . | 0.60 for albendazole [19]. |
| **Diagnostic test outcomes** |  |  |
| Variability in measured host load of infective material (eggs per examined sample of faeces) |  |  |
| *Hookworm* | Kato-Katz: negative binomial distribution with aggregation parameter $k=0.35$, estimated separately from repeated individual-level egg count data from Uganda [20]. | Kato-Katz: negative binomial distribution with aggregation parameter $k=0.35$, estimated from unpublished triple egg count data from Tamil Nadu, India |
| *Ascariasis* | Kato-Katz: negative binomial distribution with aggregation parameter $k=0.25$. | Kato-Katz: negative binomial distribution with aggregation parameter $k=0.3$ [21] |
| *Trichuris* | Kato-Katz: negative binomial distribution with aggregation parameter $k=0.25$. | Kato-Katz: negative binomial distribution with aggregation parameter $k=0.82$ [15] |
| Cut-offs for no, light, moderate, and heavy infection |  |  |
| *Hookworm* | 1, 2000, and 4000 epg | 1, 2000, and 4000 epg |
| *Ascariasis* | 1, 5000, and 50,000 epg | 1, 5000, and 50,000 epg |
| *Trichuris* | 1, 1000, and 10,000 epg | 1, 1000, and 10,000 epg |

**References**

1. Jambulingam P, Subramanian S, de Vlas SJ, Vinubala C, Stolk WA. Mathematical modelling of lymphatic filariasis elimination programmes in India: required duration of mass drug administration and post-treatment level of infection indicators. Parasit Vectors. 2016;9: 501.

2. Bradley M, Chandiwana SK, Bundy DAP, Medley GF. The epidemiology and population biology of Necator americanus infection in a rural community in Zimbabwe. Trans R Soc Trop Med Hyg. 1992;86: 73–76.

3. Elkins DB, Haswell-Elkins M, Anderson RM. The epidemiology and control of intestinal helminths in the Pulicat Lake region of Southern India. I. Study design and pre- and post-treatment observations on Ascaris lumbricoides infection. Trans R Soc Trop Med Hyg. 1986;80: 774–92.

4. Truscott JE, Turner HC, Farrell SH, Anderson RM. Soil-Transmitted Helminths: Mathematical Models of Transmission, the Impact of Mass Drug Administration and Transmission Elimination Criteria. Adv Parasitol. 2016;94: 133–198. doi:10.1016/bs.apar.2016.08.002

5. Bundy DA, Cooper ES, Thompson DE, Anderson RM, Didier JM. Age-related prevalence and intensity of Trichuris trichiura infection in a St. Lucian community. Trans R Soc Trop Med Hyg. 1987;81: 85–94.

6. Coffeng LE, Bakker R, Montresor A, de Vlas SJ. Feasibility of controlling hookworm infection through preventive chemotherapy: a simulation study using the individual-based WORMSIM modelling framework. Parasit Vectors. Parasites & Vectors; 2015;8: 541. doi:10.1186/s13071-015-1151-4

7. Bethony J, Brooker S, Albonico M, Geiger SM, Loukas A, Diemert D, et al. Soil-transmitted helminth infections: ascariasis, trichuriasis, and hookworm. Lancet. 2006;367: 1521–32. doi:10.1016/S0140-6736(06)68653-4

8. Anderson RM, Truscott J, Hollingsworth TD. The coverage and frequency of mass drug administration required to eliminate persistent transmission of soil-transmitted helminths. Philos Trans R Soc Lond B Biol Sci. 2014;369: 20130435. doi:10.1098/rstb.2013.0435

9. Truscott JE, Hollingsworth TD, Brooker SJ, Anderson RM. Can chemotherapy alone eliminate the transmission of soil transmitted helminths? Parasit Vectors. 2014;7: 266. doi:10.1186/1756-3305-7-266

10. Anderson RM, May RM. Helminth infections of humans: mathematical models, population dynamics, and control. Adv Parasitol. 1985;24: 1–101.

11. Croll NA, Anderson RM, Gyorkos TW, Ghadirian E. The population biology and control of Ascaris lumbricoides in a rural community in Iran. Trans R Soc Trop Med Hyg. 1982;76: 187–97.

12. Hotez PJ, Brooker S, Bethony JM, Bottazzi ME, Loukas A, Xiao S. Hookworm infection. N Engl J Med. 2004;351: 799–807. doi:10.1056/NEJMra032492

13. Brooker S, Bethony J, Hotez PJ. Human Hookworm Infection in the 21st Century. Adv Parasitol. 2004. pp. 197–288. doi:10.1016/S0065-308X(04)58004-1

14. Coffeng LE, Truscott JE, Farrell SH, Turner HC, Sarkar R, Kang G, et al. Comparison and validation of two mathematical models for the impact of mass drug administration on Ascaris lumbricoides and hookworm infection. Epidemics. 2017;18: 38–47.

15. Turner HC, Truscott JE, Bettis AA, Hollingsworth TD, Brooker SJ, Anderson RM. Analysis of the population-level impact of co-administering ivermectin with albendazole or mebendazole for the control and elimination of Trichuris trichiura. Parasite Epidemiol Control. 2016;1: 177–187.

16. Anderson RM, Schad GA. Hookworm burdens and faecal egg counts: an analysis of the biological basis of variation. Trans R Soc Trop Med Hyg. 1985;79: 812–825. doi:10.1016/0035-9203(85)90128-2

17. Augustine DL. Investigations on the control of hookworm disease. XVI. Length of life of hookworm larvae from the stools of different individuals. Am J Epidemiol. 1923;3: 127–136.

18. Anderson RM, May RM. Population dynamics of human helminth infections: control by chemotherapy. Nature. 1982;297: 557–563. doi:10.1038/297557a0

19. Levecke B, Montresor A, Albonico M, Ame SM, Behnke JM, Bethony JM, et al. Assessment of anthelmintic efficacy of mebendazole in school children in six countries where soil-transmitted helminths are endemic. Olliaro PL, editor. PLoS Negl Trop Dis. 2014;8: e3204. doi:10.1371/journal.pntd.0003204

20. Pullan RL, Kabatereine NB, Quinnell RJ, Brooker S. Spatial and Genetic Epidemiology of Hookworm in a Rural Community in Uganda. Bethony JM, editor. PLoS Negl Trop Dis. 2010;4: e713. doi:10.1371/journal.pntd.0000713

21. Easton AV, Oliveira RG, Walker M, O’Connell EM, Njenga SM, Mwandawiro CS, et al. Sources of variability in the measurement of Ascaris lumbricoides infection intensity by Kato-Katz and qPCR. Parasit Vectors. 2017;10: 256.
