# Supplementary material for: Sampling strategies for monitoring and evaluation of morbidity targets for soil-transmitted helminths
Source: PLoS Negl Trop Dis. 2019 Jun 26;13(6):e0007514. doi: 10.1371/journal.pntd.0007514 (PMC6615707; doi:10.1371/journal.pntd.0007514)
Supplement: S1 Fig — Prevalence is measured in all SAC living in each district at two time points: at baseline (2015) and after 5 years of PC (2020). Every randomly generated district has mean baseline prevalence between 20 and 40% (0.01 increments) (first page). School-based PC is assumed to cover children of age 2–15 (preSAC and SAC) at 75% and community-based PC is assumed to cover the entire population of age >2 at 75% (allowing for random variation in coverage between individual villages within the district). Every randomly generated district has mean baseline prevalence between 20 and 30% (second page) or 30% and 40% (third page). (PDF) [file pntd.0007514.s002.pdf]

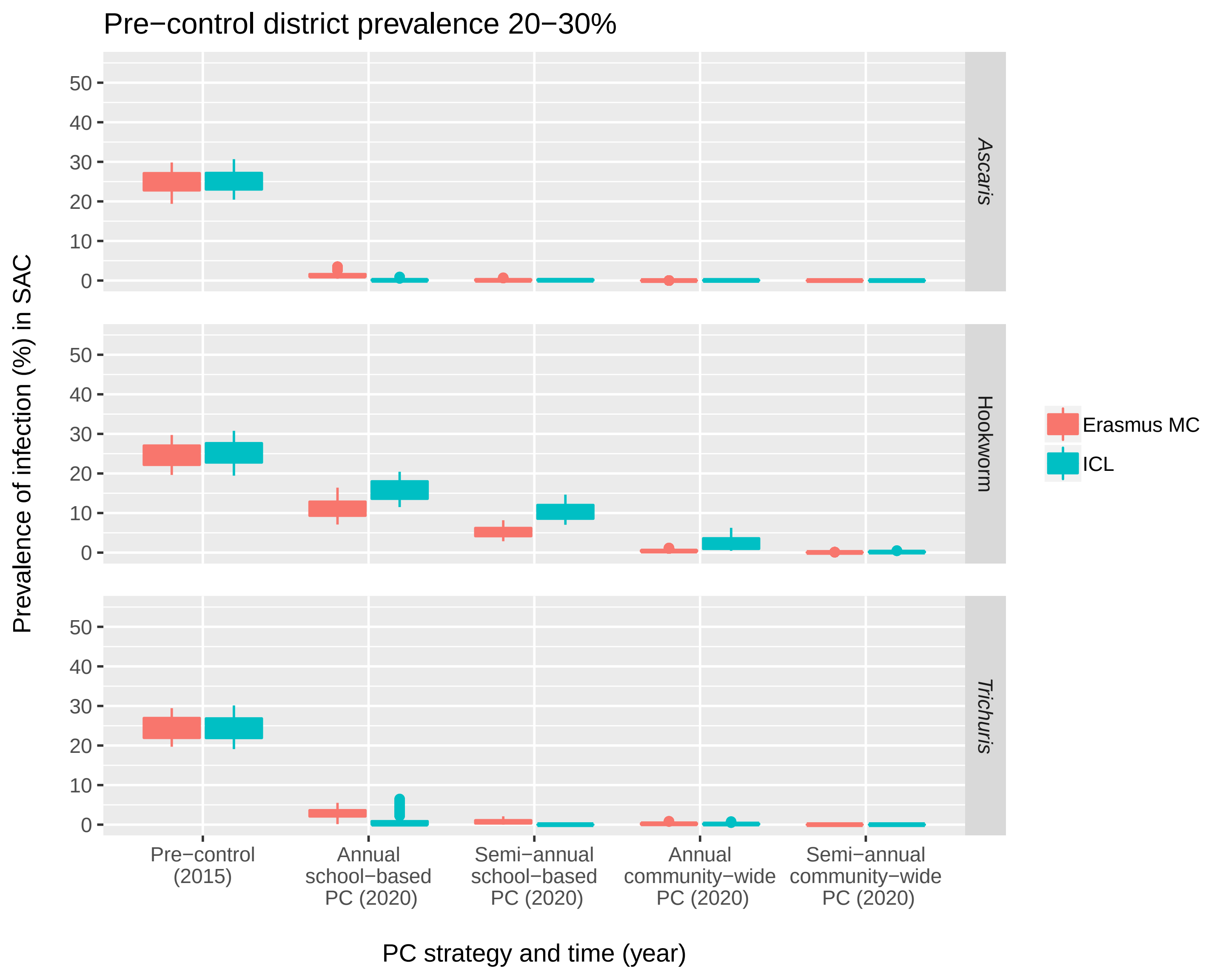

Pre−control district prevalence 20−30%
Prevalence of infection (%) in SAC
0
10
20
30
40
50
Ascaris
Hookworm
Trichuris
Erasmus MC
ICL
Pre−control (2015)
Annual school−based PC (2020)
Semi−annual school−based PC (2020)
Annual community−wide PC (2020)
Semi−annual community−wide PC (2020)
PC strategy and time (year)

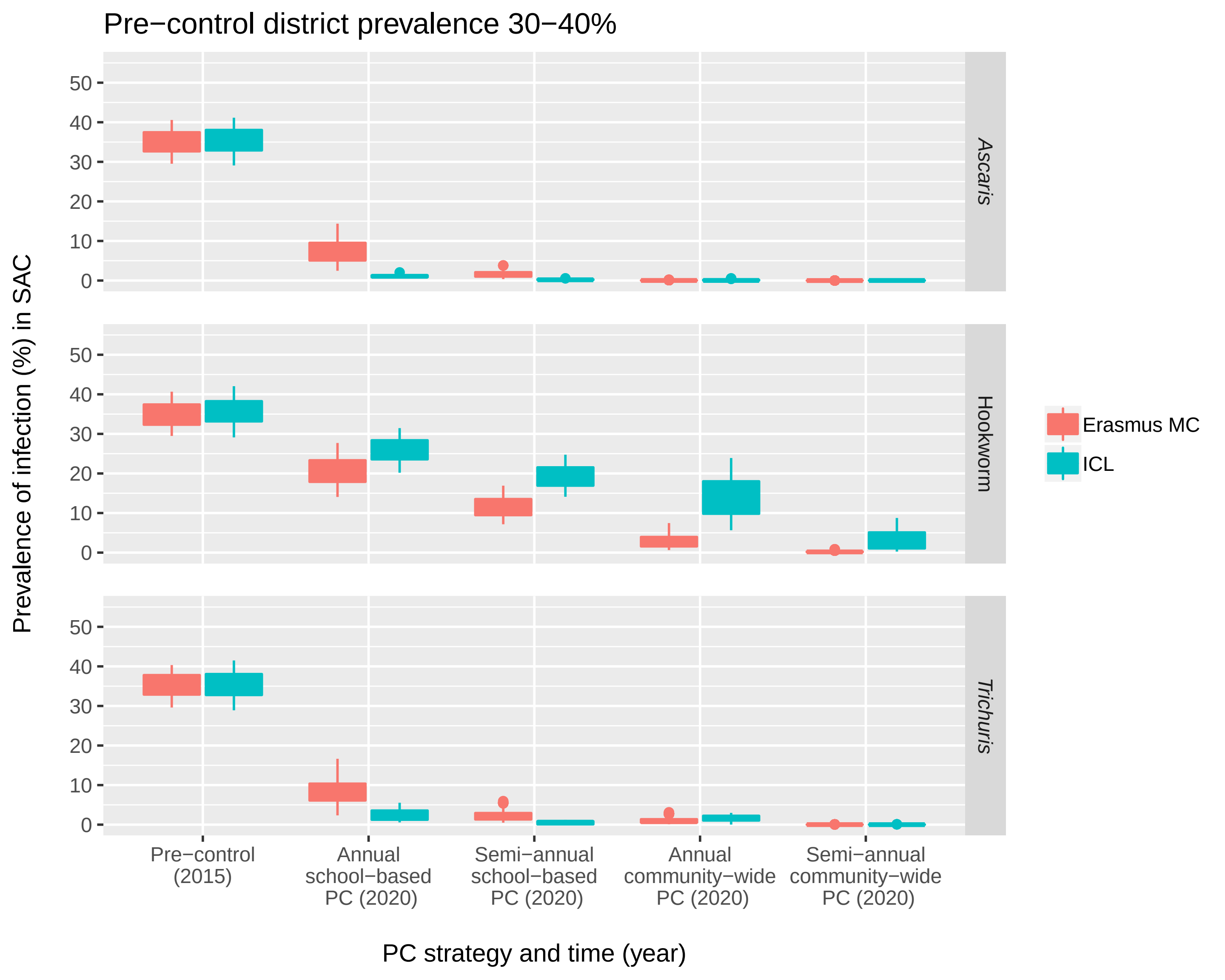
Pre−control district prevalence 30−40%
Prevalence of infection (%) in SAC
50
40
30
20
10
0
Ascaris
Hookworm
Trichuris
Erasmus MC
ICL
Pre−control (2015)
Annual school−based PC (2020)
Semi−annual school−based PC (2020)
Annual community−wide PC (2020)
Semi−annual community−wide PC (2020)
PC strategy and time (year)
