# Supplementary material for: Sampling strategies for monitoring and evaluation of morbidity targets for soil-transmitted helminths
Source: PLoS Negl Trop Dis. 2019 Jun 26;13(6):e0007514. doi: 10.1371/journal.pntd.0007514 (PMC6615707; doi:10.1371/journal.pntd.0007514)
Supplement: S2 Fig — The stacked histogram is used to distinguish between districts who have met the morbidity target in 2020 (turquoise) and those who have not (red). Meeting the morbidity target refers to reaching <1% moderate to heavy infections in all SAC in all villages (as measured by a single-slide Kato Katz). The dashed line at 1% represents the recommended threshold in prevalence of any infection among SAC required to stop PC. The numbers in grey in each panel represent the overall probability of meeting the target (i.e. the proportion of the histogram that is turquoise). Prevalence is assumed to be measured in all SAC living in randomly generated district with mean baseline prevalence ranging from 20% to 40% (first page), 20% to 30% (second page), or 30% to 40% (third page). (PDF) [file pntd.0007514.s003.pdf]

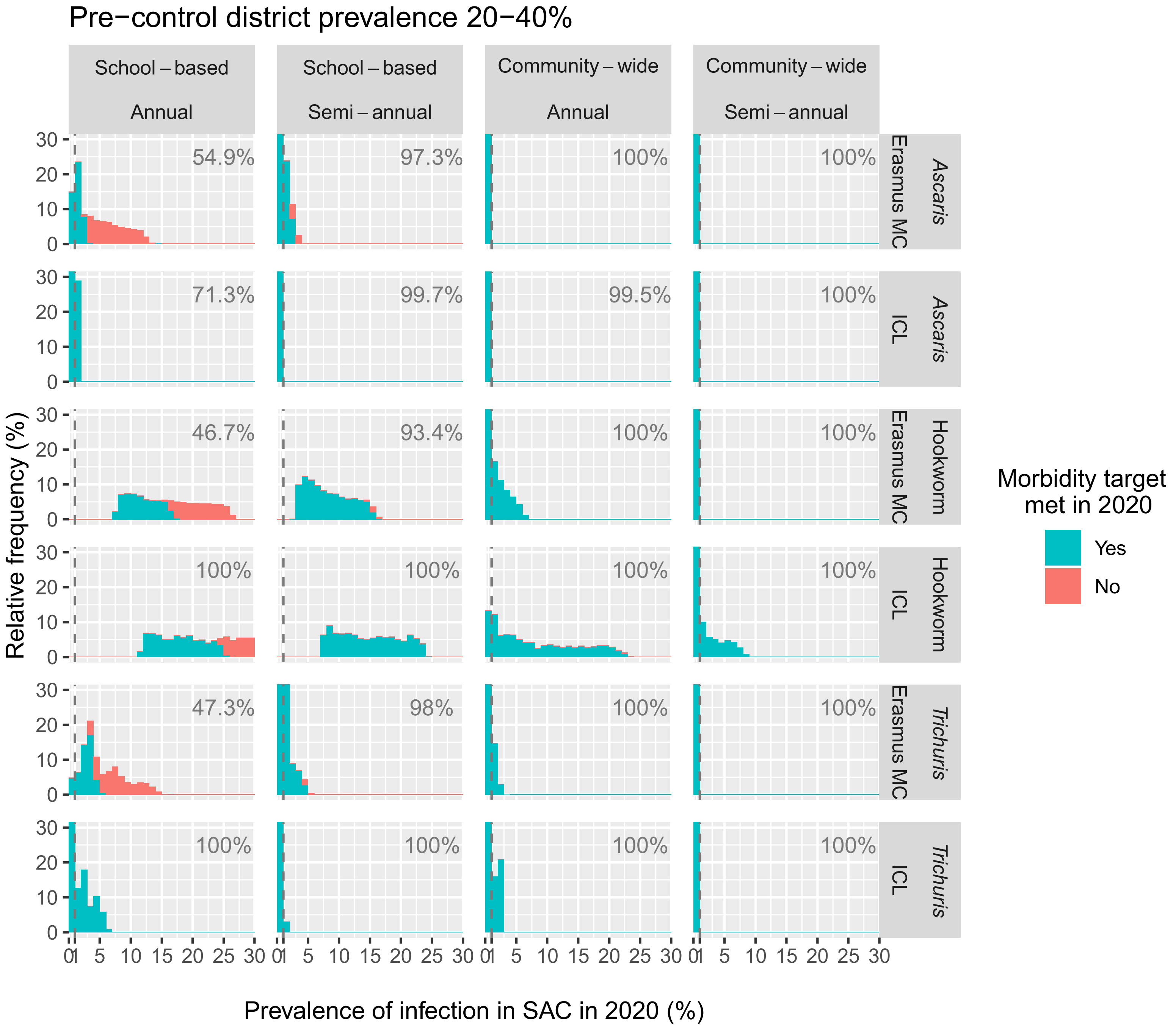

Pre−control district prevalence 20−40%
School – based Annual
School – based Semi – annual
Community – wide Annual
Community – wide Semi – annual
Erasmus MC Ascaris
ICL Ascaris
Erasmus MC Hookworm
ICL Hookworm
Erasmus MC Trichuris
ICL Trichuris
54.9%
97.3%
100%
100%
71.3%
99.7%
99.5%
100%
46.7%
93.4%
100%
100%
100%
100%
100%
100%
47.3%
98%
100%
100%
100%
100%
100%
100%
Relative frequency (%)
Prevalence of infection in SAC in 2020 (%)
Morbidity target met in 2020
Yes
No

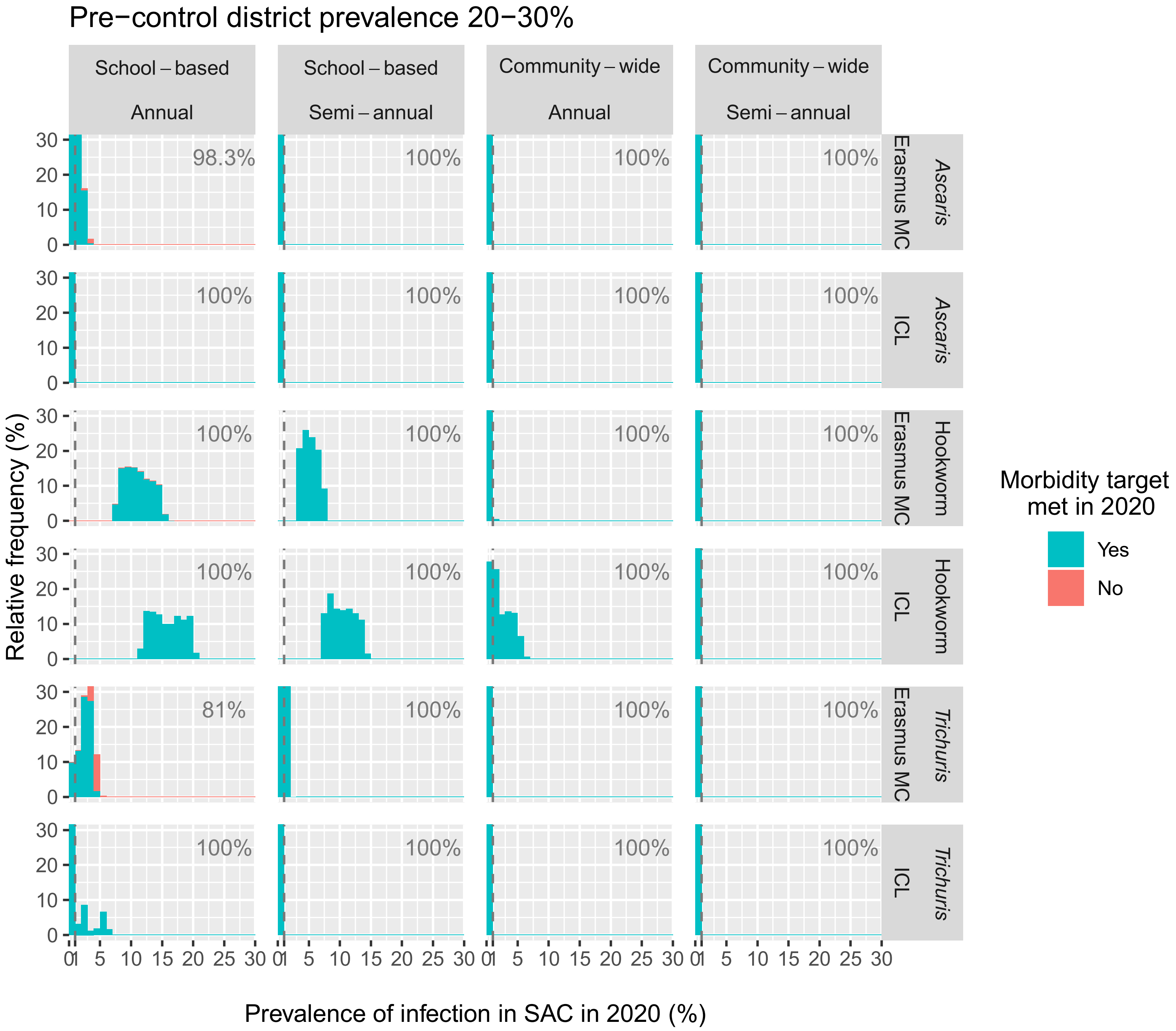
Pre−control district prevalence 20−30%
School – based
Annual
School – based
Semi – annual
Community – wide
Annual
Community – wide
Semi – annual
Ascaris
Erasmus MC
ICL
Hookworm
Trichuris
98.3%
100%
81%
Relative frequency (%)
Prevalence of infection in SAC in 2020 (%)
Morbidity target met in 2020
Yes
No

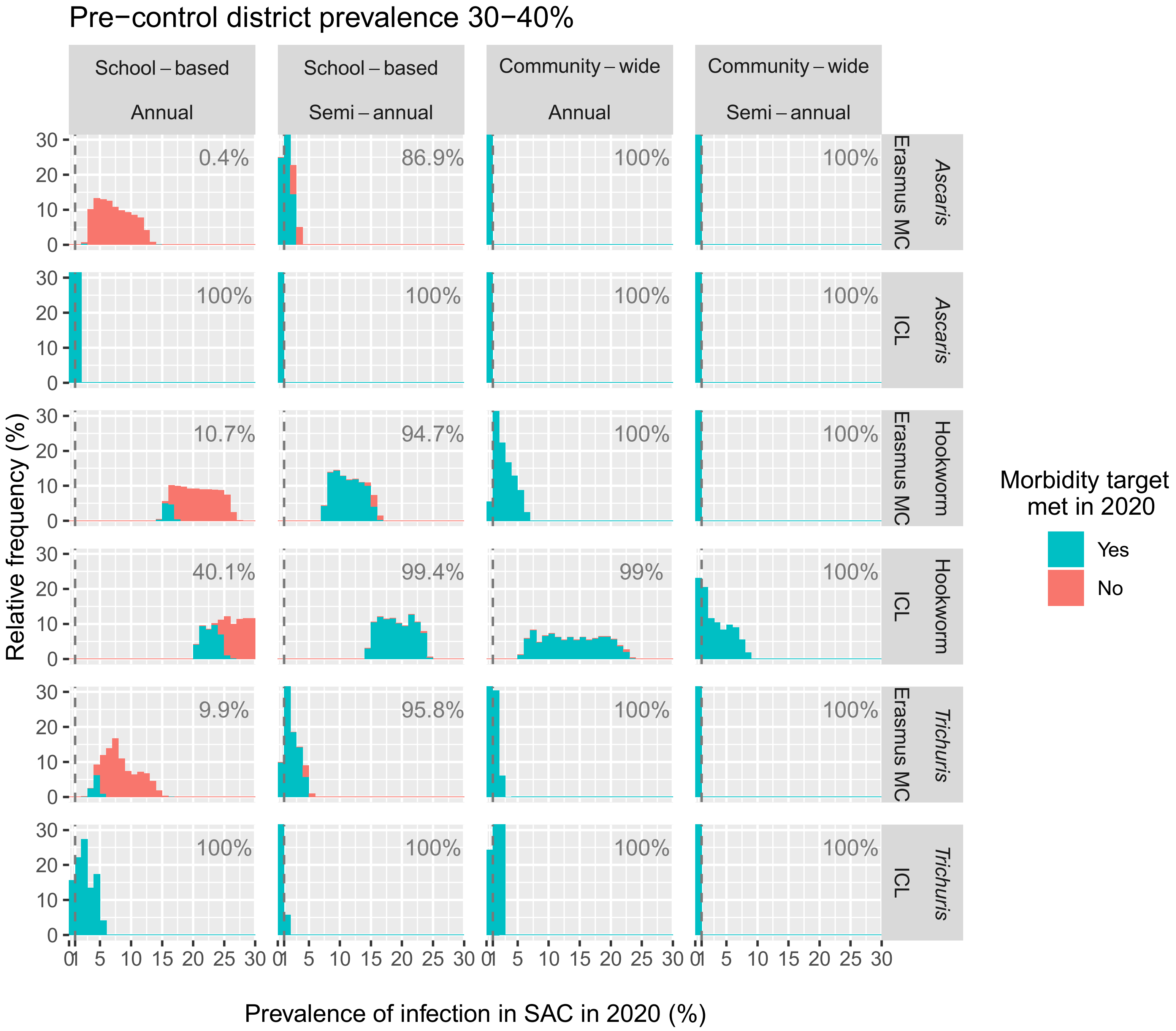
Pre−control district prevalence 30−40%
School – based Annual
School – based Semi – annual
Community – wide Annual
Community – wide Semi – annual
Ascaris Erasmus MC
Ascaris ICL
Hookworm Erasmus MC
Hookworm ICL
Trichuris Erasmus MC
Trichuris ICL
0.4%
86.9%
100%
100%
100%
100%
100%
100%
10.7%
94.7%
100%
100%
40.1%
99.4%
99%
100%
9.9%
95.8%
100%
100%
100%
100%
100%
100%
Relative frequency (%)
Prevalence of infection in SAC in 2020 (%)
Morbidity target met in 2020
Yes
No
