# Supplementary figures and images for: Sampling strategies for monitoring and evaluation of morbidity targets for soil-transmitted helminths
Source: PLoS Negl Trop Dis. 2019 Jun 26;13(6):e0007514. doi: 10.1371/journal.pntd.0007514 (PMC6615707; doi:10.1371/journal.pntd.0007514)

PPV (%) for reaching morbidity target  
in the district

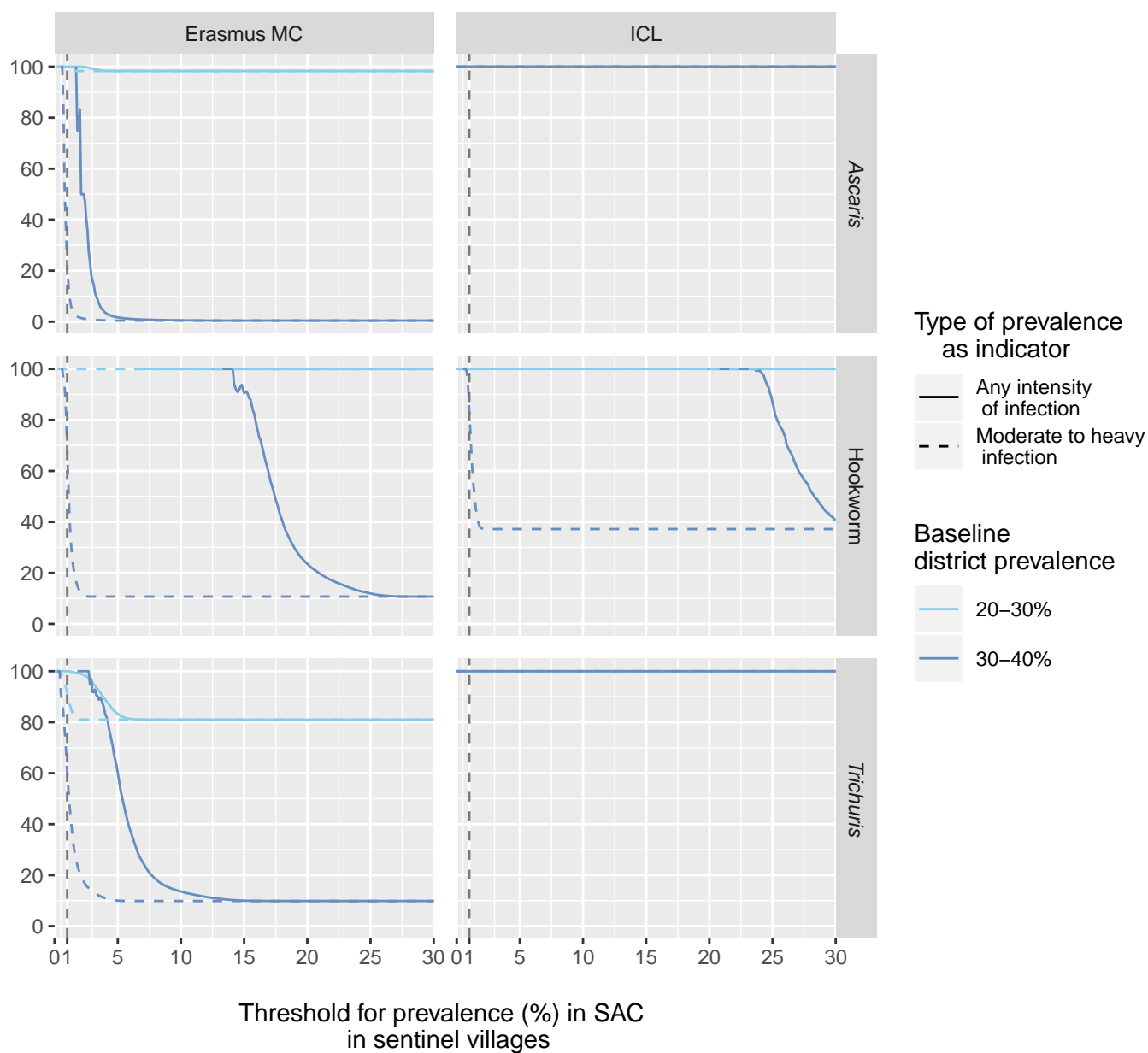

Supplement: S3 Fig — The y-axis represents the probability that the morbidity target is met in 2020 (prevalence of moderate to heavy infections in SAC <1% in the district). The x-axis represents the threshold value for prevalence of any infection or prevalence of moderate-to-heavy infections in SAC in sentinel villages, as measured with a single-slide Kato Katz. Line colours indicate results stratified by mean district baseline prevalence of infection in SAC. The line type indicates the different prevalence indicator. Only school-based annual PC is shown as more intensive PC always resulted in meeting the morbidity target. (PDF) [file pntd.0007514.s004.pdf]

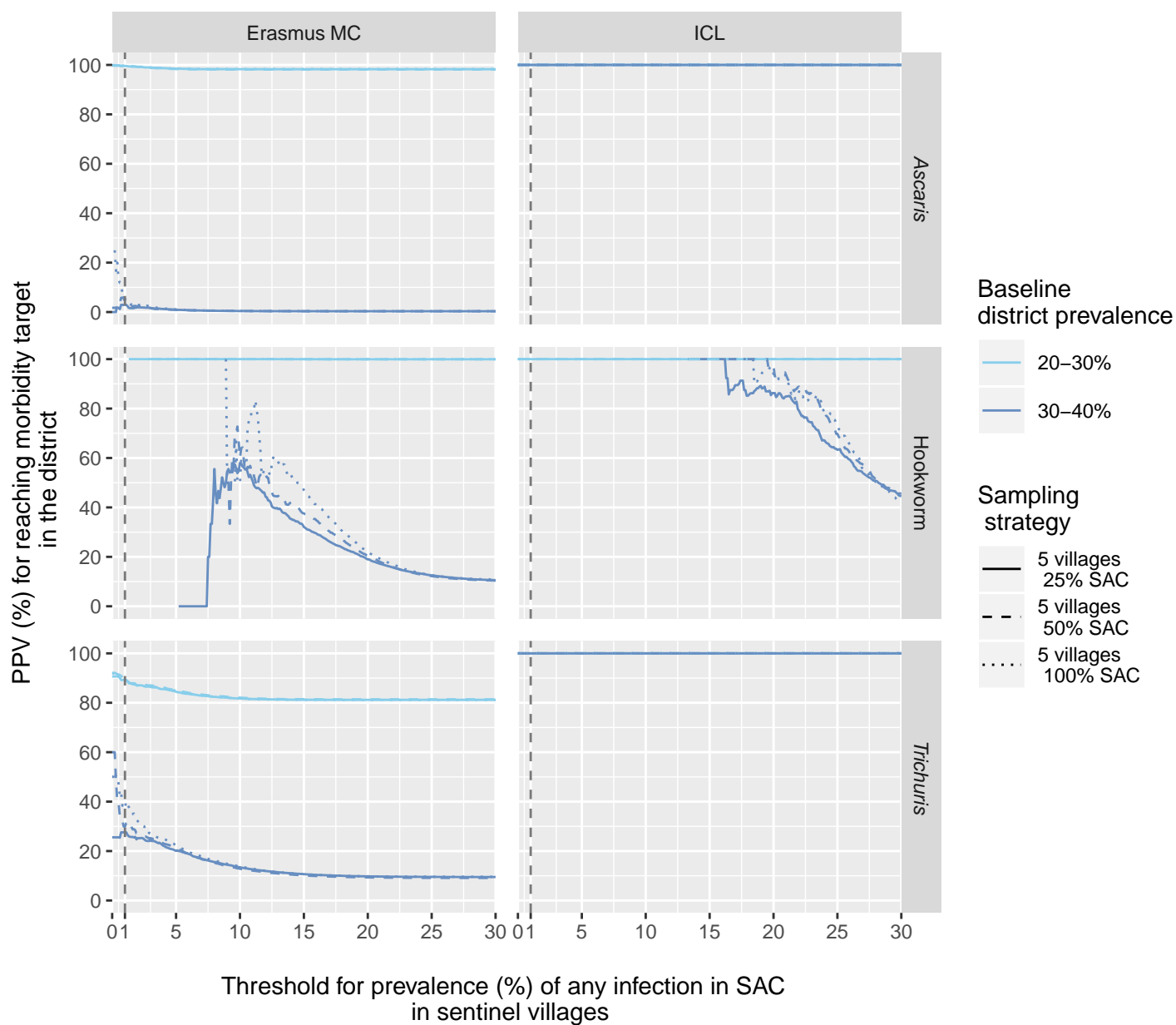

Supplement: S4 Fig — The y-axis represents the probability that the morbidity target is met in 2020 (prevalence of moderate to heavy infections in SAC <1%, averaged over all villages in the district). The x-axis represents the threshold value for prevalence of any infection in SAC in sentinel villages, as measured with a single-slide Kato Katz. Line colours indicate results stratified by mean district baseline prevalence of infection in SAC. The line type indicates different proportion of SAC sampled per village, maintaining the total number of sentinel villages constant (5). Only school-based annual PC is shown as more intensive PC always resulted in meeting the morbidity target. (PDF) [file pntd.0007514.s005.pdf]
